# Supplementary material for: Identifying candidate items for a health-related quality of life measure in young children with respiratory illness: A scoping review of generic and disease-specific measures
Source: PLoS One. 2025 May 5;20(5):e0322493. doi: 10.1371/journal.pone.0322493 (PMC12052128; doi:10.1371/journal.pone.0322493)
Supplement: S1 Appendix — (DOCX) [file pone.0322493.s001.docx]

**S3. Supplementary information 1: Full electronic search strategy for the PubMed, EBSCOhost and PsycArticles database**

Table 1 – First keyword test on PubMed

| PubMed search terms* | Number of records |
| --- | --- |
| Quality of Life"[Mesh]) AND "Process Assessment, Health Care"[Mesh]) AND "Patient Reported Outcome Measures"[Mesh]) AND "Child, Preschool" AND"chronic disease"[MeSH Terms] OR chronic illness[Mesh]. | **268,509** |
| (((("Pediatrics"[Mesh]) OR ( "Child"[Mesh] OR "Child, Preschool"[Mesh] )) AND "Quality of Life"[Mesh]) AND ( "Chronic Disease"[Mesh] OR "Noncommunicable Diseases"[Mesh] )) OR "Communicable Diseases"[Mesh] | **500,114** |
| "Child Welfare"[Mesh] OR "Child Health"[Mesh] OR "Quality of Life"[Mesh] OR QOL OR “health related quality of life” OR “health-related quality of life” OR wellbeing OR “well-being” OR HRQOL AND “Child"[Mesh] OR "Child OR “Preschool"[Mesh] OR "Pediatrics"[Mesh] OR “Infant[Mesh] OR Pediatric AND "Surveys and Questionnaires"[Mesh] OR "Patient Reported Outcome Measures"[Mesh] OR "Health Status Indicators"[Mesh] OR “instrument” OR “Scale” OR “instruments” OR Scales | **12, 030** |
| “Health Status”[Mesh] OR “quality of life”[tiab] OR “level of health” [tiab] OR “health level*”[tiab] OR “functional status”[tiab] OR “psychosocial functioning”[tiab] OR “QOL” [tiab] OR “health related quality of life”[tiab] OR “health-related quality of life”[tiab] OR “wellbeing”[tiab] OR “well-being” [tiab] OR “HRQOL”[tiab] AND "Surveys and Questionnaires"[Mesh] OR survey [tiab] OR questionnaire [tiab] OR “instrument*” [tiab] OR “instruments” [tiab] OR “Scale” [tiab] OR Scales [tiab] OR “Health Risk Appraisal*” [tiab] OR “health status indicator *”[tiab] OR “health risk appraisal*”[tiab] OR “Health Status Index”[tiab] OR “Health Status Indices”[tiab] | **64,481 results** |

**Table 2 – Final search strategy on PubMed, EBSCOhost and P tronic search strategy for the PubMed, EBSCOhost and PsycArticles database**

We searched on the PubMed, EBSCOhost and PsycArticles database using multiple keyword combinations related to health-related quality of life measures as outlined in Table 1. We limited the search to between January 2000 and November 2023 (with the most recent search conducted on 30 November 2023) and to English-language manuscripts.A

| **PubMed search terms*** |
| --- |
| (“quality of life”[Mesh] OR “QOL” OR “health related quality of life”[tiab] OR “health-related quality of life”[tiab] OR “HRQOL”[tiab] OR HRQL[tiab] OR “level of health” [tiab] OR “health level*”[tiab] OR “Health Status”[Mesh] OR “functional status”[tiab] OR “psychosocial functioning”[tiab] OR “wellbeing”[tiab] OR “well-being” [tiab] AND "Child"[Mesh] OR “Child, Preschool” [Mesh] OR "Infant"[Mesh] OR "Infant, Newborn"[Mesh] OR "Pediatrics" [Mesh] OR “Pediatric*”[tiab] OR “Child*”[tiab] OR “Infant” OR “Infants” [tiab] OR “Toddler*”[tiab] AND Surveys and Questionnaires"[Mesh] OR survey [tiab] OR questionnaire[tiab] OR “instrument” [tiab] OR “instruments” [tiab] OR “Scale” [tiab] OR Scales [tiab] OR “Health Risk Appraisal*” [tiab] OR “health status indicator *”[tiab] OR “health risk appraisal*”[tiab] OR “Health Status Index”[tiab] OR “Health Status Indices”[tiab] AND "Generic"[tw] OR "Disease-specific"[tw] OR “Self-reported”[tw] OR “Parent-proxy”[tw] OR “Self reported”[tw] OR “Parent proxy”[tw] “Develop*”[tiab] OR “Validation”[tiab]) |
| **PsycArticles search terms*** |
| "health-related quality of life" OR "Health related quality of life" OR "HRQOL" OR "HRQL" OR "Quality of life" OR "QOL" OR "Well-being" OR "Well being" OR “health status” OR “functional status” OR “psychosocial functioning” AND "questionnaires" OR "questionnaires" OR "survey" OR "surveys" OR "Instrument" OR "Instrument" or "scale" OR "Scales" OR "Health status indicator" OR "Health risk appraisal" OR "health status index" OR "Health status indices" AND "children" OR "paediatric" OR "pediatric" OR "infant*" OR "child*" AND "develop*" OR "Validation" |
| **EBSCOhost search terms *** |
| "Health-Related Quality of Life" OR "Quality of Life" OR "well-being" OR "health status" AND "children" OR "paediatric" OR "pediatric" OR "infant*" OR "child*" AND "questionnaire" OR "instrument" OR "measur*" AND "Generic" OR "Disease-specific" OR "Disease specific" OR "Parent-proxy" OR "Parent proxy" AND “Develop*” OR “Validation” |
