# Supplementary material for: Identifying candidate items for a health-related quality of life measure in young children with respiratory illness: A scoping review of generic and disease-specific measures
Source: PLoS One. 2025 May 5;20(5):e0322493. doi: 10.1371/journal.pone.0322493 (PMC12052128; doi:10.1371/journal.pone.0322493)
Supplement: Attachment — Submitted filename: 20241123_Response to reviewers_ma.docx [file pone.0322493.s003.docx]

23 November 2024

**Re: Health-related quality of life measures for children: a scoping review of generic and respiratory illness-specific measures**

Dear Editors and reviewers, PLOS One

We would like to extend our sincere gratitude to the reviewers for their constructive feedback on our manuscript. Their substantive input on the methods, findings, and discussion sections has been invaluable in enhancing the quality of our work.

Our aim with this manuscript is to chart the available generic and disease-specific health-related quality of life (HRQoL) measures for children aged 0-18-years-old. We sought to identify key components used in these HRQoL measures that could be applied for the youngest children (<5-years-old) to inform the future development of HRQoL measures for young children with respiratory illnesses living in low- and middle-income countries (LMICs). This is appropriate to a *scoping* review.

It is important to note that this review is not aimed specifically at measures for tuberculosis (TB) patients; rather, it serves as a foundational step to identify both generic measures and those specifically related to respiratory conditions. Additionally, the review is not focused solely on measures developed in LMICs, as we aim to provide a comprehensive overview of existing tools.

Thank you once again for your valuable insights, which have contributed significantly to the refinement of our manuscript. We look forward to your consideration of our revised submission.

Please find our detailed responses below.

| **#** | **Reviewer 1** | **Response** |
| --- | --- | --- |
| 1 | Abstract - Please specify the age of children in the abstract. | Thank you for your comment, we have specified the age of children in line 15-17:  Line 15-17:  The study aimed to identify key components of these measures to inform the development of a novel HRQoL tool for young children (0-5-years-old) with respiratory illnesses. |
| 2 | Background - Please justify why TB is specifically referenced in the abstract and Background. If the measure is designed for a range of respiratory illnesses then further background on this is justified. Currently, TB is the focus. Although TB is important in the SA context this may limit the applicability of a future measure in other settings. I suggest that respiratory illness be given a working definition for this review. | We are glad the reviewer highlighted this issue, as co-authors, we have also grappled with it. Respiratory illnesses generally are a significant problem for children <5-years-old, and there is a complete lack of HRQoL measures for this age group relevant to respiratory symptoms. The clinical presentation of respiratory symptoms in this age group is often similar regardless of the disease or source of illness. Our highlighting of TB in the background was as an example, using the leading cause of death by a single infectious agent with a high burden in LMICs to demonstrate the need for appropriate morbidity measures too. We have adjusted the background to clarify this. We have removed the reference to TB in the abstract.  See lines 102-148:  Lower respiratory tract infections (LRTIs) are the leading cause of paediatric hospitalisations worldwide, with an estimated 11.9 million children <5-years hospitalised in 2019 (1–5). Viral infections account for approximately 90% of all paediatric LRTIs (5). Pulmonary tuberculosis (PTB) in young children, particularly those <5-years, often presents with symptoms similar to LRTIs, making diagnosis challenging, especially in children with comorbidities such as HIV or malnutrition (6,7). In 2022, an estimated 1.3 million children aged 0-14 developed TB, yet only half received care, with the largest diagnostic gap observed in children <5-years (8). This high TB burden highlights the need to address its unique impact on children’s health and well-being. However, global data on childhood morbidity and psychosocial consequences of childhood TB remain limited (9,10).  Respiratory illnesses, including TB, can have long-term impacts on young children, extending beyond the acute phase. Early-life LRTIs are associated with later conditions such as asthma and potentially chronic obstructive pulmonary disorder (COPD) (11–13). Children with a TB history are at increased risk of lung impairment, which can have lifelong consequences (9,14,15). Despite the profound health and psychosocial implications of respiratory illnesses, current tools inadequately capture the health-related quality of life (HRQoL) impacts for young children with respiratory illnesses.  Existing data on the impact of respiratory illnesses and perceived HRQoL are particularly scarce in LMICs, where the burden of these diseases are highest. Furthermore, there are no respiratory illness HRQoL measures specifically designed for the youngest children (0-5-years). In this study, respiratory illness refers to any condition affecting the airways, lungs, or related structures, causing symptoms like coughing, wheezing, shortness of breath, or respiratory distress. This includes infections, inflammatory conditions, and structural abnormalities.  HRQoL is critical in early childhood, encompassing physical health, emotional well-being, and social interactions that are fundamental to development (16). HRQoL measures provide valuable tools for clinicians to evaluate treatments, tailor interventions, and improve care. They also inform health policy and resource allocation (16,17). These tools can be generic, applicable across populations, or disease-specific, focusing on particular symptoms or conditions (18,19). For children with respiratory illnesses, challenges such as decreased energy, sleep disturbances, and social stigma are often reported, yet no existing HRQoL measure comprehensively addresses these issues for this age group (20).  Although disease-specific HRQoL measures exist, such as CFQ-R for cystic fibrosis, PedsQL Asthma, TACQoL Asthma etc. (21–24), they are often overly specific and not suitable for general use across respiratory illnesses. Moreover, these tools have limitations when applied to high-burden, resource-constrained LMICs, where considerations of developmental milestones, cultural sensitivity, and socio-economic factors are critical.  This review provides an updated overview of HRQoL measures, with a particular emphasis on tools relevant to young children in LMICs. It builds on earlier reviews such as those by Quittner et al., (2008) and Kwon et al., 2023, by identifying critical gaps in HRQoL measurement for specific respiratory illnesses, including pulmonary TB (PTB), which is prevalent in LMICs. This study aimed to identify existing generic and disease-specific HRQoL measures for children aged 0-18-years, focusing on components relevant to children <5-years. Insights from this review will inform the development of HRQoL tools for young children with respiratory illnesses in LMICs, addressing a significant gap in the current literature.  We have also added a working definition of respiratory illness as suggested see lines 122-125:  In this study, respiratory illness refers to any condition affecting the airways, lungs, or related structures, causing symptoms like coughing, wheezing, shortness of breath, or respiratory distress. This includes infections, inflammatory conditions, and structural abnormalities. |
| 3 | Background - However, there is currently no respiratory disease-specific HRQoL measure for children.” Off the top of my head there are indeed other are other respiratory specific HRQoL measures available e.g. CFQ-R (CF), PedsQL Asthma, TACQoL Asthma, HAY. Please add this to your discussion and consider revision of the statement to imply general respiratory measures if that was implied. | You are correct and we have clarified. The statement now reads  Lines 135-139:  Although disease-specific HRQoL measures exist, such as CFQ-R for cystic fibrosis, PedsQL Asthma, TACQoL Asthma etc. (21–24), they are often overly specific and not suitable for general use across respiratory illnesses. Moreover, these tools have limitations when applied to high-burden, resource-constrained LMICs, where considerations of developmental milestones, cultural sensitivity, and socio-economic factors are critical. |
| 4 | Background - Please justify why generic and disease-specific measures for 0-18-years were reviewed when the aim was to identify key components for children 0-5 years. | While the focus of our work is indeed on children 0-5 years old, we wanted to capture any domains or items that might be relevant but are perhaps underrepresented or less explored in younger age-specific tools.  By reviewing both generic and disease-specific measures across this broader age range, we aimed to adapt and tailor concepts from older age groups to ensure no important aspects were missed for our target population. This approach allowed us to build a more comprehensive foundation, which we could then refine to suit the unique developmental and healthcare needs of younger children. |
| 5 | Background - Kindly include what additional information your review will contribute considering the review by Quittner et al in 2008 (Quittner AL, Modi A, Cruz I. Systematic review of health-related quality of life measures for children with respiratory conditions. Paediatr Respir Rev. 2008 Sep;9(3):220-32. doi: 10.1016/j.prrv.2008.05.003. Epub 2008 Jul 26. PMID: 18694714.) and Kwon et al in 2023 (Kwon, Joseph, et al. "Systematic review of the psychometric performance of generic childhood multi-attribute utility instruments." Applied Health Economics and Health Policy 21.4 (2023): 559-584.) | Thank you for suggesting these references. Both have been included: lines 140-144:  Lines 140-144:  This review provides an updated overview of HRQoL measures, with a particular emphasis on tools relevant to young children in LMICs. It builds on earlier reviews such as those by Quittner et al., (2008) and Kwon et al., 2023, by identifying critical gaps in HRQoL measurement for specific respiratory illnesses, including pulmonary TB (PTB), which is prevalent in LMICs. |
| 6 | Methods - Please justify the search terms further. Some important terms seem to be excluded e.g. outcomes, PROMs, respiratory etc | The faculty librarian and senior authors (GH & MVDZ) were consulted and assisted with refining the search terms. The keywords outlined in the methods section are only the initial search terms of our iterative search strategy (see supplementary information 1), which included a broad range of terms such as "health-related quality of life," "HRQOL," and "functional status" to capture various dimensions of quality of life assessments.  Kindly see lines 269-272 for clarity:  A faculty librarian and senior authors (GH & MVDZ) were consulted and assisted in refining the search terms. The iterative search strategy included a broad range of terms such as "health-related quality of life," "HRQOL," and "functional status" to ensure comprehensive coverage of relevant dimensions of QoL assessments. |
| 7 | Methods - Was data limited to full-text articles? | Yes, we limited our data to full-text articles. Given the scope and depth we aimed to achieve, including only full-text sources ensured we could thoroughly evaluate each study's methodology, results, and relevance. This allowed for a richer, more detailed understanding of the existing evidence, which might not have been possible with abstracts alone.  Kindly see lines 299-301:  This rigorous screening process ensured the inclusion of studies relevant to QoL in respiratory illnesses among children. Both published peer-reviewed articles and grey literature were included to provide a more comprehensive understanding of existing evidence. |
| 8 | Methods - Were the reference lists searched to identify additional publications? | Yes, absolutely! We made sure to screen the reference lists of all included articles, as well as relevant review articles, to identify any additional publications that might have been missed in the initial search. This step was invaluable in capturing related studies and ensuring a comprehensive overview for our scoping review. We have added some text to make that more clear.  Please see lines 309-386 for clarity:  Reference lists of the full-text research articles included in our analysis were also screened for additional articles to include in the review. A comprehensive review was conducted by systematically searching scientific literature, supplemented with grey literature sources to capture a broader and more inclusive range of relevant information. |
| 9 | Methods - Was the review registered in PROSPERO or another registry? | No. Though scoping reviews are still commonly not registered. This was an oversight and lesson learnt for future. |
| 10 | Methods - Please justify the age range of exclusion for ‘adolescents’ | While the WHO defines adolescence as ages 10-19-years-old, we chose to cap the age at 18 to maintain consistency with the legal age of adulthood in many settings.  See lines 172-175 for clarity:  Excluded were conceptual research, studies that applied HRQoL measures without reporting on their development or properties, review articles, and articles focused exclusively on adolescents ≥18-years or adults. The full inclusion and exclusion criteria are outlined in Table 1. |
| 11 | Methods - Please specify if software was used for data extraction and screening. | We manually extracted manuscripts into MS Excel. Although we had initially explored using Cadima (and have used it in other projects, e.g.: <https://doi.org/10.1371/journal.pgph.0000989>), we found it difficult to use for this more iterative scoping review.  See Lines 338 for more clarity on the software used for data extraction and screening:  Data extraction was conducted manually using a pre-piloted charting table in MS Excel. |
| 12 | Methods - Please justify why “articles related to diseases other than respiratory illnesses were excluded.” I assume that the development of many generic HRQoL measures did not include respiratory illness | The excluded articles encompassed a wide range of health issues, including cancers, rheumatological diseases, throat and mouth conditions, bladder and bowel disorders, bone diseases, diabetes, obesity, heart disease, and many others, such as various disabilities and skin disorders. By narrowing our scope, we aimed to capture a more cohesive understanding of HRQoL specifically in the context of respiratory illnesses, allowing us to highlight the unique challenges and needs within this population.  Please see lines 264-269:  The inclusion and exclusion criteria was refined to focus exclusively on respiratory illnesses, excluding research on other diseases and review articles. This targeted approach was intended to provide a cohesive understanding of HRQoL specifically within the context of respiratory conditions, enabling us to underscore the unique challenges and unmet needs in this population. |
| 13 | Methods - Please define respiratory illness for the review. Was a study on children with a tracheostomy and measuring health on the PTHSI or with non-invasive ventilation with muscle weakness from a neuromuscular disease included or excluded? | In our review, we defined respiratory illness as any condition affecting the airways, lungs, or related structures that results in symptoms such as coughing, wheezing, shortness of breath, or respiratory distress. This definition encompasses a range of issues, including infections, inflammatory conditions, and structural abnormalities. Please see response to point 2 above.  Regarding the inclusion criteria, we excluded studies focusing on cases requiring mechanical intervention or presenting with severe respiratory compromise, which includes children with tracheostomies and those using non-invasive ventilation due to muscle weakness from neuromuscular diseases. By doing so, we aimed to concentrate on respiratory conditions that are more common and not affected by other issues such as muscle weakness allowing for a clearer analysis of HRQoL in this specific context. |
| 14 | Methods - Please specify what ‘Google search engine’ refers to. | We included a more general search using the google web browser  See lines 303:  A grey literature search was conducted using the Google web browser. |
| 15 | Methods - What QoL websites were searched and/or how were they determined? What about conference proceedings. | We did explore various QoL websites during our search. These were: EORTC Quality of life ( https://qol.eortc.org/), international society of quality of life research ( <https://www.isoqol.org/)Pedsql> ( <https://eprovide.mapi-trust.org/instruments/pediatric-quality-of-life-inventory/pedsql-review-copies> etc. However, we encountered several with errors or outdated information, which led us to prioritise peer-reviewed journal articles instead. By focusing on these credible sources, we aimed to ensure the scientific rigour and reliability of the evidence included in our review.  As for conference proceedings, we did not screen those for this particular review. While they can provide valuable insights, we wanted to maintain a consistent standard of evidence, and peer-reviewed articles offered the robustness we were looking for. We recognise this as a limitation and have included it in our study discussion.  See line 749-752 on the limitations of not including QoL websites and conference proceedings:  Additionally, the exclusion of HRQoL websites and conference proceedings may have omitted emerging tools or insights not yet published in peer-reviewed literature. Consequently, some recent developments in HRQoL measures for paediatric respiratory conditions may not be fully captured in our analysis. |
| 16 | Methods - Data extraction – who piloted the charting/data extraction table. Please specify domains – was this a domain which may have consisted of additional items e.g. Physical functioning or were domains and individual items extracted? | The data extraction process was piloted by the first and second. We structured our extraction headings to capture a comprehensive view, including whether the measure was generic or disease-specific, the type of disease if it was disease-specific, whether it was a parent proxy measure or a self-report, and the domains measured.  See lines 386-397:  Data extraction was conducted manually using a pre-piloted charting table in MS Excel.  The charting table included data on the study authors and year of publication, generic or disease-specific measures, domains measured, target population (including age), self-report or parent proxy report, response options, and language.  The researchers intentionally did not start with a predefined list of domains, as they wanted to be as comprehensive as possible and allow for the natural emergence of relevant domains during extraction. After gathering the data, we grouped the individual domains based on related attributes—for example, all aspects related to mobility, such as physical functioning, running, and walking, were categorised under the broader physical domain. This approach allowed us to maintain rigour and ensure that our findings captured the full breadth of relevant measures, with the senior authors verifying our groupings to uphold the study's integrity. |
| 17 | Methods - Please describe how age ranges were categorized for this manuscript. | We categorised the age ranges in our manuscript to specifically focus on children aged 0-5-years-old, as this is the primary demographic of interest. To maintain relevance and accuracy, we highlighted articles that catered exclusively to this age group, avoiding older ages because developmental stages vary significantly, and the items or domains relevant to older children may not apply to those 0-5-years-old.  In our scoping review, we organised the age ranges based on the measures themselves. In the table, we first showcased the 0-5-year-old category, followed by the 4-9-year-old group, and finally included broader ranges such as 2-18-year-olds. This structured approach allowed us to group the age ranges from youngest to oldest, ensuring clarity in how the measures were categorised.  See lines 425-427 for clarity:  Measures were also grouped based on the applicable age range with tools for children 0-5-years priortised in the presentation. |
| 18 | Results - HRQOL Domains and items In general, the domains are reflective of the definition of HRQoL. Please justify why items were not reviewed by domain if your aim was to identify what would be important for a new measure. | Yes, we were looking for a HRQoL measure, so this would reflect the definitions of HRQoL. Our goal in the review is to chart the available resources to identify potential gaps. We believe that this is what we have achieved. In separate work, we have engaged what domains are applicable to respiratory illnesses and the youngest children (<https://doi.org/10.1186/s12955-024-02308-0>; and https://doi.org/10.5588/pha.22.0038) |
| 19 | Results - I am not sure that your analysis of lated versions is correct – This may be included in studies that you have excluded. E.g. PedsQl generic measure is validated and translated into South African languages. Consider omitting data on translations. | To clarify, we did not analyse translated versions. We only included these in the table for readers’ reference. We have now excluded these as per your suggestion. |
| 20 | Results - The data extraction is currently limited and does not address consistency of use, definitions and criteria which are alluded to in the discussion. | The intent in this scoping review is to chart the available tools. This includes some messiness and lack of consistency of definition in those tools. We have attempted to be as inclusive as possible. |
| 21 | Discussion - “There has also been 231 limited development or validation of available measures in LMIC, where the socio-economic 232 context and the burden of respiratory diseases is different.” Your study was not designed to discuss the validation of measures in LMIC. These may have been applied but were excluded in your review. If the intention is to motivate for a new measure in a LMIC considering our burden of disease, then this needs to be discussed in more detail in the background. This is currently only related to TB. Consider adding discussion of the SDGs and other global health initiatives. | We clarify that this point in the discussion to show an ongoing limitation of available HRQoL measures for youngest children.  As suggested, we have added detail / amended the background to highlight that the overwhelming burden of respiratory illness in children is experienced in LMIC and we therefore need HRQoL tools that are designed for optimal use in such contexts. Please refer to comment 2. |
| 22 | Discussion - It is unclear how the considerations for age-specific developmental milestones is concluded from your results. | We agree, we have removed this point. |
| 23 | Discussion - Please justify “most 243 HRQoL measures were originally developed in HICs and cannot be translated into other 244 settings without considering the different socioeconomic contexts”. In general guidelines suggest that there is cultural adaptation and translation when outcome measures are used in different contexts. This has been done successfully with many instruments. | While guidelines do recommend cultural adaptation, this also has limits and can be imperfect. We will clarify this point in the manuscript to emphasise the complexities involved in adapting HRQoL measures for use in LMICs, alongside acknowledging the successful adaptations that have occurred for some instruments. Thank you for prompting us to refine this discussion!  See lines 700-712 for clarity:  Adapting HRQoL measures for LMIC contexts involves addressing linguistic, cultural, and developmental variations, as well as differing health beliefs and socioeconomic challenges (84,85). This process requires translation, back-translation, and cultural validation to ensure tools are relevant and meaningful for local populations. Collaboration with local experts, caregivers, and communities is crucial to ensuring the content reflects socio-cultural norms and values. For instance, domains like social functioning or emotional well-being may need redefinition or adaptation for cultural resonance (86).  Research has demonstrated that HRQoL tools like the PedsQL or EQ-5D-Y can be successfully adapted for LMICs through rigorous cultural validation and iterative testing, such as cognitive interviews and pilot studies. These adaptations underscore the importance of contextualiSed approaches in HRQoL measurement. However, the lack of a disease-specific HRQoL measure tailored to young children with respiratory illnesses in LMICs—especially TB—remains a critical gap (86–88). |
| 24 | Discussion - Observable behaviours are included in your discussion. It is currently not obviously recorded in the results. | We agree, we have removed this point. |
| 25 | Discussion - Further discussion points such as ‘a new measure should include extended social support networks and socioeconomic context and factors that are significant in LMICs’ are quite general and sweeping. This extraction of this data from the existing measures would strengthen the review. | We have removed this point. |
| 26 | General - Please consider revising for academic writing. I suggest that this includes less reference to ‘we’ | We have revised the manuscript accordingly. |
|  | **Reviewer 2 feedback** | **Response** |
| 27 | There appears to be a disconnect between the background and the research questions. While the research aim, as the title suggests, is to chart HRQoL measures for children broadly, the study's initial focus is on TB patients in low- and middle-income countries, which feels more specific. I feel either the authors need to revise the title and the research aims or they should provide more comprehensive context on the existing HRQoL measures—specifically, which are the most commonly used generic and respiratory illness-specific measures for children, and how have they been applied in previous research? | Thank you for this insightful comment! I appreciate your attention to ensuring clarity between the background and research questions.  Our goal was indeed broader: To map available HRQoL measures, both generic and respiratory-specific, to identify key components that could inform the development of a novel HRQoL tool for young children with respiratory illnesses—including TB—in LMICs. The intention was not to focus solely on TB or limit the review to measures developed in LMICs, but rather to provide a comprehensive overview of what's currently available and how these tools are used in paediatric respiratory contexts.  We have clarified in comment 2. |
| 28 | The author mentioned “there is currently no respiratory disease-specific HRQoL measure for children”, I do not think this is true, as this study was about to identifying these measures | We have provided clarity in this regard in a previous comment.  Please also see response to comment 2 and 3. |
| 29 | It is also unclear to me that, how non-TB disease-specific instruments will contribute to assessing HRQoL in TB patients. The authors may need to either expand in the background to explain why they included respiratory illness instruments in general or narrow their focus to TB-specific or generic instruments that are used in TB patients. | Our focus is on respiratory illnesses among youngest children, not TB. We have clarified this in the text. Please also see response to comment 3. |
| 30 | I have significant concerns about the search strategy employed, which casts doubt on the comprehensiveness of the results. the authors should provide the search terms in the appendix for transparency. Given the broad scope of the search, it is surprising that only 1,823 papers were identified. This relatively small yield suggests the search may not have been as exhaustive as intended. I recommend that the search strategy be re-evaluated to ensure it is capturing all relevant literature. | Please see response to comment 6. |
| 31 | The exclusion criteria, particularly for generic measures, are unclear. It is not clear how the authors can identify generic measures for use in children with respiratory illness, without looking at those paper “assessing the quality of life using the measure”. A clearer outline of these criteria is necessary to justify the results. | We appreciate the opportunity to clarify our approach! Our review did not apply exclusion criteria for generic HRQoL measures, as our aim was to explore a broad range of instruments used to assess quality of life in children with respiratory illnesses. Our inclusion criteria allowed us to capture studies that either described, developed, or evaluated HRQoL measures for children aged 0-18-years-old, including both generic and respiratory-disease-specific tools. To ensure consistency, we excluded articles describing translations of a measure, as well as disease-specific tools unrelated to respiratory illnesses. |
| 32 | Upon reviewing the keywords, it seems no terms specifically related to respiratory illnesses were included. This omission likely reduces the search’s effectiveness in capturing relevant studies. Additionally, the use of terms like "develop" and "validation" may have inadvertently excluded valuable studies that assess instruments but do not focus on their development or validation. | Yes, there is a breadth of literature available of studies reporting HRQoL in populations *using* these tools. But we were interested in the tools themselves. It’s true that any assessment of a tool (i.e., after its development and validation) may have been excluded from our initial round of searching. But (a) these would likely have been found the next steps of searching reference lists of included tools, and (b) are not strictly necessary to the stated purpose of charting the available tools / landscape of HRQoL measurement. Scoping reviews are not about capturing every use of a tool, but rather to say what the outline of available measures looks like in overview. We did not include respiratory specific terms in our search to ensure to capture both generic as well as disease specific tools (excluding non-respiratory tools). |
| 33 | the authors limited their search to studies published between January 2000 and November 2023. Many widely used generic HRQoL measures were developed before 2000, so this time restriction may exclude important foundational studies. I therefore have major concern on this timeframe. | We set our timeframe from 2000 onward to focus on more recent developments in HRQoL measurement, as our review aims to map tools that have been evaluated or updated in the context of current health needs and standards. However, we understand that some foundational HRQoL measures widely used today were indeed developed prior to 2000, and their exclusion may have limited our review. We note this in the limitations. However, we stand by the principle that a HRQoL tool that has not been adapted / updated since 2000 is likely of less relevance to novel tools specific to youngest children with respiratory illness.  We have included a line in the limitation section to highlight the potential limitation of only including articles from the year 2000. See lines 747-753:  Additionally, limiting the review to English-language articles may have resulted in the omission of relevant research conducted in other languages, particularly from LMICs where diverse cultural and contextual factors significantly impact children's HRQoL. As a result, this may affect the comprehensiveness of our findings and limit the generalisability of the conclusions drawn from the available literature. |
| 34 | The manuscript does not include quality assessment of the reviewed studies or HRQoL instruments. Have lall the included instruments been validated? A formal quality assessment framework is essential in systematic reviews. | This is a scoping review, not a systematic review. The point of quality assessments in systematic reviews is because the intention is to pool across studies. This does not apply in scoping reviews where the intention is to chart the breadth of available data / measures. |
| 35 | It would be helpful to include a frequency chart showing how often each domain (e.g., physical, emotional, social functioning) is covered by the identified generic and disease-specific instruments. This would allow readers to better understand which areas of HRQoL are well-represented and mostly related to the diseases. | Thank you for this excellent suggestion. We completely agree that a frequency chart would enhance the understanding of how various domains are represented across the identified instruments.  We have included a frequency graph in the revised manuscript, highlighting the coverage of each domain across the measures. You can find it in lines 521 and 530 (Separate document S2- Domains and Dimesions). |
| 36 | Providing detailed information about the specific items included in the various instruments would further improve the manuscript’s utility. This would give readers a better understanding of which aspects of HRQoL are being measured by each instrument, and could help guide future research or clinical applications. | In this *scoping* review, we are looking to chart the landscape of available measures, not items within those measures. In companion work we have address this comment somewhat – <https://doi.org/10.1186/s12955-024-02308-0> |
|  | **Reviewer 3 feedback** | **Response** |
| 37 | Clarity of the background: In general, I think the authors need to organizing the background more clearly so that the reader can understand the depth and the impact of your study. As it stands, the rationale for the study is challenging to follow, which may hinder readers’ understanding of its significance. | We appreciate your insights on enhancing the clarity of our background section. Please see response to comment 2 above. |
| 38 | I question the authors statement that  “our understanding of the impact of respiratory illnesses, including TB, on the HRQoL of children, both during the disease episode and beyond treatment completion, is extremely limited.”  There is a considerable body of research on TB, asthma, and other respiratory illnesses. The authors should provide a stronger argument for why a scoping study is necessary to develop a new tool in this field. | Thank you for your thoughtful critique. We appreciate your point about the existing body of research on respiratory illnesses, including TB and asthma. However, we disagree that there is a considerable body of work on the impact of respiratory illnesses on HRQoL, especially in young children living in LMICs.  We used our scoping review to identify existing measures, thereby laying the groundwork for developing a novel HRQoL measure that is sensitive to the specific needs and experiences of young children with respiratory conditions in LMIC settings.  We have rephrased Lines 119-120 as follows:  Existing data on the impact of respiratory illnesses and perceived HRQoL are particularly scarce in LMICs, where the burden of these diseases are highest. |
| 39 | What do you mean by ‘there is no respiratory disease specific hrqol measure for children’? In your study itself, you have 21 studies about this topic. | When we stated that "there is no respiratory disease-specific HRQoL measure for children," we were emphasising the lack of tools specifically designed for children with a broad range of respiratory illnesses. While our review indeed identified 21 studies addressing HRQoL in this context, most of these measures focus on one specific type of respiratory illness. To be more specific we have corrected our statement to read “there is no respiratory illness HRQoL measure for youngest children (0-5-years-old). The only HRQoL measure explicitly designed for 0-5-year-olds that we found pertains to cystic fibrosis, which does not adequately address the unique needs and experiences of children with TB or other respiratory conditions. Our goal is to highlight these gaps to motivate the development of a comprehensive HRQoL measure tailored for young children facing various respiratory illnesses, including TB.  See lines 121-122:  " Furthermore, there are no respiratory illness HRQoL measures specifically designed for the youngest children (0-5-years). |
| 40 | Line 127  What do you mean by QoL websites? Is there any? | When we referred to "quality of life (QoL) websites," we meant online resources dedicated to HRQoL assessment tools, guidelines, and research related to various health conditions. For example, the EORTC Quality of Life website (https://qol.eortc.org/) and the EuroQol website (https://euroqol.org/) serve as valuable repositories for QoL measures and instruments. |
| 41 | Line 127  What is the authors consideration by using Google scholar as the primary tool for literature searches? | We did not use Google Scholar as the primary tool for our literature searches. Instead, we consulted with our faculty librarian, who recommended specific databases tailored for our review. We focused on three key databases to ensure a comprehensive and rigorous search strategy.  These databases were chosen for their relevance and ability to capture high-quality literature in the field.  See lines 261-269 on the databases used:  An initial search of PubMed was conducted to identify relevant articles. This involved analysing the title and abstract text as well as the MeSH terms used to index the articles. Used to describe the articles. Based on these findings, the search terms were refined to focus on quality of life (QoL) and children. The inclusion and exclusion criteria was refined to focus exclusively on respiratory illnesses, excluding research on other diseases and review articles. This targeted approach was intended to provide a cohesive understanding of HRQoL specifically within the context of respiratory conditions, enabling us to underscore the unique challenges and unmet needs in this population. A revised search was undertaken across PubMed, EBSCOhost and PsycArticles. |
| 42 | Do you think involving grey literature may affect the quality of your study? Please put this in the discussion section. | We believe that including grey literature actually enhances the quality of our study. Our search for grey literature revealed several peer-reviewed articles that were not identified in the database searches, providing a more comprehensive view of the available HRQoL measures. This broader perspective helps to address potential gaps in the literature and contributes valuable insights to our findings.  We have added lines 303-384 for clarity:  A grey literature search was conducted using the Google web browser. Keywords and inclusion criteria were applied, and results were limited to the first ten pages of search results. A screening process similar to that of the database searches was followed. Reference lists of systematic reviews and included full-text articles were manually screened for additional studies.  Reference lists of systematic reviews and articles that were identified through the database search and Google search were manually screened. Reference lists of the full-text research articles included in our analysis were also screened for additional articles to include in the review. A comprehensive review was conducted by systematically searching scientific literature, supplemented with grey literature sources to capture a broader and more inclusive range of relevant information. |
| 43 | Line 160  What do you mean by discrete measures? | In this context, we are referring to HRQoL measures that are distinct and specific, typically focusing on particular domains or aspects of HRQoL rather than providing a comprehensive assessment. These discrete measures allow researchers and clinicians to pinpoint specific areas of concern or interest, facilitating targeted interventions and evaluations. However, we have deleted the phrase discrete from the text.  We have revised Line 440-444 as follows:  In total, 72 research articles met the inclusion criteria and were included in the full-text review. These studies described 41 HRQoL measures, including 20 generic and 21 disease-specific tools. Among these, five measures (four generic and one disease-specific measure—the Cystic Fibrosis Questionnaire-Revised) were developed specifically for children aged 0–5 years (Tables 2 and 3). |
| 44 | Table 2:  a. Please put the table heading on each page  b. Remove the phrase ‘years old’  c. Decrease the font size for better fit  d. Remove the terms ‘domain’, and ‘items’  e. Remove the phrase ‘Measurement’, retain the scale  f. Add a column to indicate whether the measure has been validated in the LMIC (yes/no) | We have amended the table as recommended please see Lines 630-647 |
| 45 | Discussion - IN the first paragraph, authors should summarize the main findings that address the research question. In your study, I suppose that the study aim is the key component of HRQoL instrument for children. | We have added a paragraph to the discussion to highlight the summarise the main findings please see Lines 653-661.  Lines 653-661:  This review screened 1,823 peer-reviewed articles, ultimately including 72 studies reporting on 41 distinct HRQoL measures, of which 20 were generic and 21 were disease-specific. Notably, only five measures—four generic and one disease-specific (for cystic fibrosis)—were designed for children aged 0-5-years. The identified HRQoL measures displayed substantial variability, encompassing 101 domains across generic measures and 79 across disease-specific measures. While the most commonly assessed domains included physical and emotional well-being, the majority of measures were developed in HICs, with only two originating from LMICs. This underscores a significant gap in contextually relevant HRQoL tools for children with respiratory illnesses, including PTB, in LMIC settings. |
|  | **Reviewer 4 feedback** | **Response** |
| 46 | While I support researchers to develop new instrument, it is important to acknowledge the limitations of existing instruments. In other words, if no studies have demonstrated the lack of validity and sensitivity of existing instruments, why would the authors consider developing a new instrument in first place? On the one hand, a newly developed instrument may have a better sensitivity, on the other hand, this may hamper comparison across studies if everyone is using a different instrument. | Thank you for your insightful feedback. We wholeheartedly agree that it is essential to acknowledge the limitations of existing HRQoL instruments and also the importance of comparison across studies. We are hoping to address exactly that issue. To have a HRQoL tool for youngest children (0-5-years-old) that is general enough to be applicable to a range of respiratory illnesses that it can be used across diseases and settings, but still specific to respiratory illnesses and not so generic as to be only the most basic level of HRQoL generally. With the aim to have a tool that can be used widely to ensure comparison across studies. |
| 47 | While the manuscript provides a detailed list of HRQoL measures and their dimensions, there is some inconsistency in defining and categorizing domains across different instruments. Consider using a standardized framework to group these dimensions for easier comparison. | We appreciate your observation regarding the inconsistency in defining and categorising domains across the various HRQoL measures. This is an inherent problem with the available HRQoL tools. We have chosen to report the domains as used by the tools included rather than recategorize these based on our judgement. |
| 48 | The manuscript highlights the need for a new HRQoL tool for young children in LMICs but does not provide specific guidance on how this new tool should differ from existing ones. Including a discussion on specific attributes (e.g., developmental milestones, socio-economic context, or caregiver input) that should be emphasized would strengthen the practical utility of the review. Also, I am not sure about how a HRQoL tool in LMICs could differ with the HRQoL tool used in developed countries. Can you clarify? | We agree that outlining specific attributes for this tool would enhance its practical utility. Our review emphasises that the new measure should incorporate essential aspects such as childhood developmental milestones, extended social support networks, and the unique socioeconomic contexts of LMICs. Furthermore, it should assess child-specific HRQoL by capturing observable changes in behavior and other relevant factors.  We have included specific guidance on what the new tool should include in lines 700-706:  Adapting HRQoL measures for LMIC contexts involves addressing linguistic, cultural, and developmental variations, as well as differing health beliefs and socioeconomic challenges (84,85). This process requires translation, back-translation, and cultural validation to ensure tools are relevant and meaningful for local populations. Collaboration with local experts, caregivers, and communities is crucial to ensuring the content reflects socio-cultural norms and values. For instance, domains like social functioning or emotional well-being may need redefinition or adaptation for cultural resonance (86). |
| 49 | The exclusion of studies published before 2000 and those not in English is reasonable given resource constraints. However, briefly acknowledging potential biases or limitations this might introduce would add transparency to the review process. This could be a big limitation considering the aim is to develop a HRQoL tool for LMICs. I could imagine many studies from LMICs are published in non-English journals? | We appreciate your recognition of the rationale behind excluding studies published before 2000 and those not in English, especially given our resource constraints. We have added some clarity on the limitations in lines 745-753  Lines 745-753:  A comprehensive search strategy captured all available data on HRQoL measures for children, providing a valuable resource for researchers. However, restricting the search to English-language articles published from 2000 onward may have excluded foundational studies or non-English research, particularly from LMICs. Additionally, limiting the review to English-language articles may have resulted in the omission of relevant research conducted in other languages, particularly from LMICs where diverse cultural and contextual factors significantly impact children's HRQoL. As a result, this may affect the comprehensiveness of our findings and limit the generalisability of the conclusions drawn from the available literature. |
|  | In the key words used for this scoping review, I do not see the word “LMICs”. This seems inconsistent between the scoping review and the motivation of conducting this study. | We have addressed this comment in a previous comment. Please refer to comment 32 |

Sincerely,

Michaile Gizelle Anthony on behalf of the co-authors

Tel: +27 671850267

e-mail: manthony@sun.ac.za
