# Supplementary material for: Identifying candidate items for a health-related quality of life measure in young children with respiratory illness: A scoping review of generic and disease-specific measures
Source: PLoS One. 2025 May 5;20(5):e0322493. doi: 10.1371/journal.pone.0322493 (PMC12052128; doi:10.1371/journal.pone.0322493)
Supplement: Attachment — Submitted filename: 20250303_Response to reviewers_ma.docx [file pone.0322493.s004.docx]

03 March 2025

**Re: Health-related quality of life measures for children: a scoping review of generic and respiratory illness-specific measures**

Dear Editors and reviewers, PLOS One

We would like to extend our sincere gratitude to the reviewers for their constructive feedback on our manuscript.

Our aim with this manuscript is to chart the available generic and disease-specific health-related quality of life (HRQoL) measures for children aged 0-18-years-old. We sought to identify key components used in these HRQoL measures that could be applied for the youngest children (<5-years-old) to inform the future development of HRQoL measures for young children with respiratory illnesses living in low- and middle-income countries (LMICs). This is appropriate to a *scoping* review.

Thank you once again for your valuable insights, which have contributed significantly to the refinement of our manuscript. We look forward to your consideration of our revised submission.

Please find our detailed responses below.

|  | **Editor** | **Response** |
| --- | --- | --- |
| **1** | As reviewer #1 points out, the manuscript has improved substantially, but there are still quite a few problematic issues to address. Please take all comments raised by the reviewer into account and revise accordingly. | Thank you for your comment. We have taken the comments from reviewer 1 into account and have revised the manuscript accordingly |
| **#** | **Reviewer 1** | **Response** |
| 1 | I agree with reviewer 2 that the title and aim of the systematic review could be changed to better clarify your aim. It appears that the aim of your review is in fact to identify candidate items to include in a new measure of (HRQoL) for children living with respiratory illness in a LMIC setting. I suggest that your review is revised to clarify this and the results and discussion should better reflect this.  This review has not critically appraised the measures or assessed their suitability for use in a LMIC nor within general respiratory illness. | Thank you. As noted, this was a scoping review, and our aim was indeed to identify candidate items for a new health-related quality of life (HRQoL) measure for young children with respiratory illnesses in a low- and middle-income country (LMIC) setting.  To ensure our objectives are communicated clearly, we have revised the title of the manuscript as follows:  “Identifying candidate items for a health-related quality of life measure in young children with respiratory Illness: A scoping review of generic and disease-specific measures”.  We have also have also revised the aim of the scoping review to maintain clarity and to explicitly reflect the focus on identifying candidate items:  See lines 16-18:  “To identify candidate items from existing HRQoL measures to inform the development of a new HRQoL tool for young children (0-5 years-old) with respiratory illnesses in a LMIC setting”  As this was a scoping review rather than a systematic review, its purpose was to map existing HRQoL measures and extract relevant candidate items rather than critically appraise their validity or suitability for use in an LMIC or respiratory illness context.  To ensure this distinction is clear, we have explicitly stated in the methods section that our review followed an established scoping review methodology, which does not include a critical appraisal of included measures. |
| 2 | Despite the authors stating that the reference to TB had been removed from the abstract, it in fact has not. | We have removed the reference to TB in the abstract  See Lines 11- 12:  Health-related quality of life (HRQoL) in young children affected by respiratory illnesses remains understudied, particularly in low- and middle-income countries (LMICs), where the burden of these diseases is disproportionately high |
| 3 | The updated text justifying the need to specifically reference TB has lead to further ambiguities.  The authors second sentence states that Viral infections account for 90% of all paediatric LRTIs which, to me, would imply that this would be the most important consideration for developing a respiratory HRQoL measure.  Line 75-77 state that only half of the 1.3 million children received care with the diagnostic gap largest in those < 5 years – this indicates a need for improved diagnostic and treatment packages and not necessarily a HRQoL measure.  The emphasis on TB is still incredibly large considering that this measure is intended to be used as a general measure for a variety of respiratory illnesses. | We appreciate the reviewer’s feedback regarding the emphasis on TB in the introduction and the potential ambiguities it introduced. To address this concern, we have removed all references to TB and revised the introduction to ensure a broader focus on respiratory illnesses as a whole.  Kindly see lines 63-139:  Lower respiratory tract infections (LRTIs) are the leading cause of paediatric hospitalisations worldwide, with an estimated 11.9 million children <5-years hospitalised in 2019 (1–5). Viral infections account for approximately 90% of all paediatric LRTIs (5), but bacterial infections, inflammatory conditions, and structural abnormalities also contribute to significant morbidity. The burden of these illnesses is particularly high in low- and middle-income countries (LMICs), where access to healthcare, early diagnosis, and appropriate management can be challenging (5).  Respiratory illnesses can have long-term impacts on young children, extending beyond the acute phase. Early-life LRTIs are associated with later conditions such as asthma and potentially chronic obstructive pulmonary disorder (COPD). Children who experience recurrent or severe respiratory illnesses may face ongoing respiratory symptoms, developmental delays, and psychosocial challenges, affecting their overall well-being (6–8). Existing data on the impact of respiratory illnesses and perceived HRQoL are particularly scarce in LMICs, where the burden of these diseases is highest (9–11). In this study, respiratory illness refers to any condition affecting the airways, lungs, or related structures, causing symptoms like coughing, wheezing, shortness of breath, or respiratory distress. This includes infections, inflammatory conditions, and structural abnormalities.  Despite the profound health and social implications of respiratory illnesses, current tools do not capture their impact on young children's health-related quality of life (HRQoL) (9–11). HRQoL is a multidimensional concept that encompasses physical health, emotional well-being, and social interactions, all of which are critical for early childhood development (12).  Standard clinical assessments often focus on disease severity and treatment outcomes but do not comprehensively measure how these conditions affect a child's daily life, functioning, and overall well-being.  There is a notable gap in HRQoL measurement for young children with respiratory illnesses, particularly in LMICs, where the burden is highest. While some disease-specific HRQoL measures exist—such as those for asthma and cystic fibrosis. HRQoL measures provide valuable tools for clinicians to evaluate treatments, tailor interventions, and improve care. They also inform health policy and resource allocation (12,13). These tools can be generic, applicable across populations, or disease-specific, focusing on particular symptoms or conditions (14,15). For children with respiratory illnesses, challenges such as decreased energy, sleep disturbances, and social stigma are often reported (9), yet no existing HRQoL measure comprehensively addresses these issues for this age group (9,10).  Although disease-specific HRQoL measures exist, such as CFQ-R for cystic fibrosis, PedsQL Asthma, TACQoL Asthma etc. (16–19), they are often overly specific and not suitable for general use across respiratory illnesses (11). Moreover, these tools have limitations when applied to high-burden, resource-constrained LMICs, where considerations of developmental  milestones, cultural sensitivity, and socio-economic factors are critical (10,11).  This review provides an updated overview of HRQoL measures, with a particular emphasis  on tools relevant to young children in LMICs. It builds on earlier reviews such as those by  Quittner et al., (2008) and Kwon et al., 2023, by identifying critical gaps in HRQoL measurement for specific respiratory illnesses, including pulmonary TB (PTB), which is  prevalent in LMICs. These critical gaps include the focus on young children (0-5-years-old), identifying gaps in existing HRQoL measures, addressing the LMIC as well as highlighting the need for a comprehensive approach to the development of HRQoL measure for young children 0-5-years-old in LMICs.  This study aimed to identify candidate items from existing HRQoL measures to inform the development of a new HRQoL tool for young children (0-5 years-old) with respiratory illnesses in a LMIC setting, addressing a significant gap in the current literature. |
| 4 | Line 84-86 requires a reference. | Thank you for catching that. We have gone ahead and double-checked the text, and we have included reference for these lines.  Kindly see lines 80-81 in the revised manuscript:  Despite the profound health and social implications of respiratory illnesses, current tools do not capture their impact on young children's health-related quality of life (HRQoL) (9–11).  References used:  Anthony MG, Hoddinott G, Van Niekerk M, Mtshabe A, Pienaar A, Dewandel I, et al. Caregivers’ perspectives on health-related quality of life in young children with TB and respiratory illnesses. Public Heal Action [Internet]. 2022;12(1):201–5. Available from: http://dx.doi.org/10.5588/pha.16.0125%0A  Anthony, M. G., Van Niekerk, M., Hesseling, A. C., Hoddinott, G., & van der Zalm, M. M. (2024). An item bank to measure health-related quality of life among young children (0-5-years-old) affected by respiratory illnesses - expert stakeholders and end-users from the Western Cape, South Africa. Health and quality of life outcomes, 22(1), 95. <https://doi.org/10.1186/s12955-024-02308-0>  Anthony, M. G., Johnson, L. S., van Niekerk, M., Mfwaze, A., Bavuma, B., Hesseling, A. C., Hoddinott, G., & van der Zalm, M. M. (2024). Health-related quality of life in children with presumptive TB. IJTLD open, 1(11), 495–500. <https://doi.org/10.5588/ijtldopen.24.0355> |
| 5 | Line 86-89 requires a reference. | We have gone ahead and double-checked the text, and we have included reference for these lines.  Kindly see lines 75-76:  Existing data on the impact of respiratory illnesses and perceived HRQoL are particularly scarce in LMICs, where the burden of these diseases is highest (9–11). |
| 6 | Reference 20 can be used to describe the challenges of decreased energy, sleep disturbances and social stigma but NOT that no existing HRQoL measure addresses these issues. A new reference should be added to defend the second half of this sentence. | We have included a reference to defend the 2^nd^ half of the sentence as follows:  See lines 118-120:  For children with respiratory illnesses, challenges such as decreased energy, sleep disturbances, and social stigma are often reported (11), yet no existing HRQoL measure comprehensively addresses these issues for this age group (9,11).  Reference used:  Anthony, M. G., Johnson, L. S., van Niekerk, M., Mfwaze, A., Bavuma, B., Hesseling, A. C., Hoddinott, G., & van der Zalm, M. M. (2024). Health-related quality of life in children with presumptive TB. IJTLD open, 1(11), 495–500. https://doi.org/10.5588/ijtldopen.24.0355 |
| 7 | Please add a reference for line 104-105 “they are often overly specific and not suitable for general use across respiratory illness” | We have included a reference for line 121-123:    Although disease-specific HRQoL measures exist, such as CFQ-R for cystic fibrosis, PedsQL Asthma, TACQoL Asthma etc. (17–20), they are often overly specific and not suitable for general use across respiratory illnesses (10).  Reference used:  Anthony, M. G., Van Niekerk, M., Hesseling, A. C., Hoddinott, G., & van der Zalm, M. M. (2024). An item bank to measure health-related quality of life among young children (0-5-years-old) affected by respiratory illnesses - expert stakeholders and end-users from the Western Cape, South Africa. Health and quality of life outcomes, 22(1), 95. https://doi.org/10.1186/s12955-024-02308-0 |
| 8 | Please add a reference to substantiate the argument in line 105-107. | We have included a reference to substantiate lines 105-107:  See lines: 123-125  Moreover, these tools have limitations when applied to high-burden, resource-constrained LMICs, where considerations of developmental  milestones, cultural sensitivity, and socio-economic factors are critical (9,10).  References used:  Anthony, M. G., Van Niekerk, M., Hesseling, A. C., Hoddinott, G., & van der Zalm, M. M. (2024). An item bank to measure health-related quality of life among young children (0-5-years-old) affected by respiratory illnesses - expert stakeholders and end-users from the Western Cape, South Africa. Health and quality of life outcomes, 22(1), 95. <https://doi.org/10.1186/s12955-024-02308-0>  Anthony, M. G., Johnson, L. S., van Niekerk, M., Mfwaze, A., Bavuma, B., Hesseling, A. C., Hoddinott, G., & van der Zalm, M. M. (2024). Health-related quality of life in children with presumptive TB. IJTLD open, 1(11), 495–500. <https://doi.org/10.5588/ijtldopen.24.0355> |
| 9 | Please expand on what is meant by ‘critical gaps’ | Thank you for your feedback. We acknowledge the need for clarity regarding the contribution of this scoping review and have refined the text to better articulate the critical gaps it addresses.  This review fills key gaps in the literature by:  1. Focusing on young children (0–5-Years): While previous reviews (e.g., Quittner et al., 2008; Kwon et al., 2023) have examined HRQoL measures for paediatric respiratory illnesses, they primarily focus on older children or specific conditions. Our review specifically addresses the youngest age group, where HRQoL measurement is particularly challenging due to limited verbal communication and developmental variability.  2. Identifying limitations of existing measures: We highlight that while several disease-specific HRQoL tools exist (e.g., CFQ-R for cystic fibrosis, PedsQL Asthma), they are often highly specific and do not adequately capture the broader impact of respiratory illnesses. Additionally, many of these tools were developed in high-income settings and may not be directly applicable to LMICs without adaptation.  3. Addressing the LMIC context: Respiratory illnesses in LMICs occur within unique socio-economic, cultural, and healthcare contexts. Our review underscores the lack of HRQoL tools that consider factors such as poverty, access to care, malnutrition, and social support—elements that can significantly shape a child's experience of illness and recovery.  4. Highlighting the need for a comprehensive approach: Unlike prior reviews that focus on a single disease or symptom domain, our review synthesises evidence across multiple respiratory illnesses, advocating for a more integrated approach to HRQoL measurement.  Kindly see lines 170-177:  This review provides an updated overview of HRQoL measures, with a particular emphasis  on tools relevant to young children in LMICs. It builds on earlier reviews such as those by  Quittner et al., (2008) and Kwon et al., 2023, by identifying critical gaps in HRQoL  measurement for specific respiratory illnesses, including pulmonary TB (PTB), which is  prevalent in LMICs. These critical gaps include the focus on young children (0-5-years-old), identifying gaps in existing HRQoL measures, addressing the LMIC as well as highlighting the need for a comprehensive approach to the development of HRQoL measure for young children 0-5-years-old in LMICs. |
| 10 | If the aim of your research is to identify items that may be relevant to include in the development of a new measure then I would suggest that this be made clearer. | We have revised the aim as follows:  See lines 134 -135:  “To identify candidate items from existing HRQoL measures to inform the development of a new HRQoL tool for young children (0-5 years-old) with respiratory illnesses in a LMIC setting” |
| 11 | Inclusion criteria states that articles that evaluated the psychometric properties of HRQoL measures was included – this is not evident from your results or reference list. | Thank you for your careful review and for highlighting this inconsistency. We have removed the statement regarding the inclusion of studies assessing psychometric properties, as this was not the primary objective of our scoping review. Instead, our review aimed to identify candidate items from existing HRQoL measures to inform the development of a new tool for young children (0–5-years old) with respiratory illnesses in an LMIC setting.  Our focus was on mapping the content of available HRQoL measures rather than evaluating their psychometric properties. While some of the included studies may have reported psychometric data, this was not a requirement for inclusion, nor was it a central aspect of our analysis. We have revised the text to ensure alignment between our objectives, methods, and reported findings. |
| 12 | Previous comment 7 : Methods – was data limited to full-text articles has not been addressed. Authors response “This rigorous screening process ensured the inclusion of studies relevant to QoL in respiratory illnesses among children. Both published peer reviewed articles and grey literature were included to provide a more comprehensive understanding of existing evidence.” Does not refer to full-text articles nor is this included in the inclusion/exclusion criteria. | Thank you for your follow-up on this point. To clarify, our review was indeed limited to full-text, peer-reviewed articles. This applies both to studies identified through database searches and to grey literature, which consisted of full-text, peer-reviewed articles that were not captured in traditional database searches but were relevant to our review.  We have removed the following text from the inclusion criteria see Table 1 line 175:  Research articles that evaluated the psychometric properties of HRQoL measures.  We have also reworked lines 206-210 to include clarity regarding the full-text peer reviewed articles:  This rigorous screening process ensured that only full-text, peer-reviewed articles were included in the scoping review. Additionally, grey literature was limited to full-text, peer-reviewed articles that were not identified through database searches, ensuring a comprehensive synthesis of the existing evidence on QoL in respiratory illnesses among children. |
| 13 | Your response to previous comment 13 is appreciated. It is however not addressed in the manuscript. Please ensure that these details are captured in the text or in your table of inclusion and exclusion criteria. | We have added the following text to the exclusion criteria see Table 1 line 175:  Research articles focusing on cases requiring mechanical intervention or presenting with severe respiratory compromise, which includes children with tracheostomies and those using non-invasive ventilation due to muscle weakness from neuromuscular diseases were excluded. |
| 14 | Findings line 295 please specify the four generic measures identified for children aged 0-5 years. | We have included the four generic measures for young children which includes: IQI, TAPQOL, EQ-TIPS and ITQOL-SF-47  See lines 267-268:  20 generic and 21 disease-specific tools. Among these, five measures (four generic—IQI, TAPQOL, EQ-TIPS and ITQOL-SF-47nd one  disease-specific measure—the Cystic Fibrosis Questionnaire-Revised) were developed  specifically for children aged 0–5 years (Tables 2 and 3). |
| 15 | Findings – kindly note that the TANDI has been renamed the EQ-TIPS and should be referenced as such. | Thank you for pointing this out we have renamed the TANDI to EQ-TIPS throughout the manuscript. |
| 16 | Findings age range – the PedsQL has developed specific versions for children aged between 0-5 years. The TAPQoL, ITQol, C-QoL, HSCS-PS (HuPs). Please refer to Kwon – I believe that there are close to 20 versions available for children <5 years. | Thank you for your thoughtful comment and for highlighting these additional measures. We appreciate your observation regarding the PedsQL. While we acknowledge that the PedsQL covers a broad age range (0–18-years), it does not offer a dedicated measure specifically tailored for children aged 0–5-years, which remains a critical gap in existing HRQoL tools.  In line with your suggestion, we have already included the TAPQoL and ITQoL in our review, as both measures address aspects of HRQoL for young children. However, the C-QoL and HSCS-PS (HuPs) did not emerge in our database searches despite our comprehensive screening process and were therefore not included. Your suggestion to refer to Kwon’s work is appreciated, and we have cross-referenced this to strengthen the comprehensiveness of our manuscript. |
| 17 | Line 338 “One measure did not report 338 specific domains” please specify what measure this is” | We have revised the sentence as follows see lines 294-295:  One measure namely the Exeter health related quality of life measure (EXQOL) did not report on specific domains |
| 18 | Line 368. The development of the EQ-5D-Y took place in multiple countries, including SA, it was not just validated in a LMIC. | We have revised the sentence as follows see lines 316-318:  The development of the EQ-5D-Y took place in multiple countries, including SA, however, was not validated in a LMIC. |
| 19 | Discussion line 420 – “There is a paucity of HRQoL measures—both generic and disease-specific—specifically designed for children aged 0-5-years, a group disproportionately affected by respiratory illnesses”  This statement is quite controversial and I do not think that it is a finding of your study. As you did not include the performance of generic measures in respiratory disease or appraise the psychometric performance of these measures in respiratory disease nor LMIC nor 0-5 years of age. | We have removed this sentence from the discussion. |
| 20 | Existing tools exhibit inconsistent definitions, criteria, and inclusion of domains and items, limiting comparability across studies. Your results section does not address the definitions applied to these measures. Your study did not appraise the domains, items and comparability of instrument findings. | Thank you for your insightful comment. We acknowledge that our study didn not critically appraise the domains, items and comparability of instrument findings. We have rephrased this more carefully.  “ While this was not a systematic review and we did not critically appraise the domains, items and comparability of instruments we did note inconsistencies in them limiting comparability between studies when assessing HRQoL. |
| 21 | I agree that most HRQoL measures were developed in HICs. This review cannot substantiate that these measures are not readily adaptable to LMICs without accounting for distinct cultural, linguistic and socioeconomic factors. Your search criteria nor your results allow this discussion nor this conclusion. | We appreciate your observation that the review, as currently presented, does not adequately address the cultural, linguistic, and socioeconomic factors that may impact the adaptability of HRQoL measures developed in HICs to LMICs.  We acknowledge that without a thorough examination of these factors, it is premature to definitively conclude that existing measures are not readily adaptable to LMIC contexts. Our initial intention was to highlight the limitations of applying HIC-based HRQoL tools in LMICs, based on the differences in healthcare systems, socioeconomic conditions, and cultural contexts. However, we agree that our current search criteria and results do not provide sufficient evidence to substantiate this claim.  In response to your comment, we have included lines 363-373 to the discussion to more carefully consider the complexities of cross-context applicability.  See lines 360-369:  While HRQoL measures developed in HICs may not fully account for the unique challenges faced in LMICs, further research is necessary to assess the influence of cultural, linguistic, and socioeconomic factors on their applicability. |
| 22 | Lines 433-439 and 440-442 are important to note. It is not clear that this was the aim nor the focus of this literature review. | We have removed lines 433-439:  Adapting HRQoL measures for LMIC contexts involves addressing linguistic, cultural, and developmental variations, as well as differing health beliefs and socioeconomic challenges (79,80). This process requires translation, back-translation, and cultural validation to ensure tools are relevant and meaningful for local populations. Collaboration with local experts, caregivers, and communities is crucial to ensuring the content reflects socio-cultural norms and values. For instance, domains like social functioning or emotional well-being may need redefinition or adaptation for cultural resonance (11).  And we have also removed lines 440-442 to reduce ambiguities regarding the aim of the study:  Research has demonstrated that HRQoL tools like the PedsQL or EQ-5D-Y can be successfully adapted for LMICs through rigorous cultural validation and iterative testing, such as cognitive interviews and pilot studies. These adaptations underscore the importance of contextualised approaches in HRQoL measurement. However, the lack of a disease-specific HRQoL measure tailored to young children with respiratory illnesses in LMICs—especially TB—remains a critical gap (14,84,85). |

Sincerely,

Michaile Gizelle Anthony on behalf of the co-authors

Tel: +27 671850267

e-mail: manthony@sun.ac.za
